# Stochastic Spatial Heterogeneity in Activities of H^+^-ATP-Ases in Electrically Connected Plant Cells Decreases Threshold for Cooling-Induced Electrical Responses

**DOI:** 10.3390/ijms22158254

**Published:** 2021-07-31

**Authors:** Ekaterina Sukhova, Daria Ratnitsyna, Vladimir Sukhov

**Affiliations:** Department of Biophysics, N.I. Lobachevsky State University of Nizhny Novgorod, 603950 Nizhny Novgorod, Russia; n.catherine@inbox.ru (E.S.); dasha-lola1997@mail.ru (D.R.)

**Keywords:** H^+^-ATP-ase, plant electrical responses, stochastic spatial heterogeneity, diversity-induced resonance, cooling, simulation

## Abstract

H^+^-ATP-ases, which support proton efflux through the plasma membrane, are key molecular transporters for electrogenesis in cells of higher plants. Initial activities of the transporters can influence the thresholds of generation of electrical responses induced by stressors and modify other parameters of these responses. Previously, it was theoretically shown that the stochastic heterogeneity of individual cell thresholds for electrical responses in a system of electrically connected neuronal cells can decrease the total threshold of the system (“diversity-induced resonance”, DIR). In the current work, we tested a hypothesis about decreasing the thresholds of generation of cooling-induced electrical responses in a system of electrically connected plant cells with increasing stochastic spatial heterogeny in the initial activities of H^+^-ATP-ases in these cells. A two-dimensional model of the system of electrically connected excitable cells (simple imitation of plant leaf), which was based on a model previously developed in our works, was used for the present investigation. Simulation showed that increasing dispersion in the distribution of initial activities of H^+^-ATP-ases between cells decreased the thresholds of generation of cooling-induced electrical responses. In addition, the increasing weakly influenced the amplitudes of electrical responses. Additional analysis showed two different mechanisms of the revealed effect. The increasing spatial heterogeneity in activities of H^+^-ATP-ases induced a weak positive shift of the membrane potential at rest. The shift decreased the threshold of electrical response generation. However, the decreased threshold induced by increasing the H^+^-ATP-ase activity heterogeneity was also observed after the elimination of the positive shift. The result showed that the “DIR-like” mechanism also participated in the revealed effect. Finally, we showed that the standard deviation of the membrane potentials before the induction of action potentials could be used for the estimation of thresholds of cooling-induced plant electrical responses. Thus, spatial heterogeneity in the initial activities of H^+^-ATP-ases can be a new regulatory mechanism influencing the generation of electrical responses in plants under actions of stressors.

## 1. Introduction

H^+^-ATP-ases, which are a P-type ATPases, support active proton efflux through the plasma membrane in cells of plants and fungi [1,2,3]. The molecular transporters can be considered as the main active electrogenic transporters in the plant plasma membrane [4,5], participating in the secondary active transport of inorganic ions and organic compounds (e.g., sugars or amino acids), plant tolerance to salt stress, stomata opening and plant movement, regulation of intracellular pH and pH signaling, cellular expansion and acid growth, etc.

H^+^-ATP-ases play an important role in the forming of plant electrical responses, which are local or propagating changes in electrical potential in the plasma membrane and induced by actions of stressors [6,7,8,9,10,11,12,13,14]. It should be noted that there are several types of electrical responses in higher plants including (i) local electrical responses, which are generated in the irritated zone [15,16,17]; (ii) action potentials, which are induced by weak or moderate irritations (e.g., cooling or touch) and actively propagate through the plant body [6,7,8]; (iii) variations potentials, which are probably local electrical responses on the propagation of hydraulic and/or chemical signals after action of local damages (e.g., burning, heating, and crush) [7,8,9,10,11,12,13,14,18,19,20,21,22,23,24]; and (iv) system potentials, which are weakly investigated hyperpolarization signals [10,11,12,13,25,26]. The electrical responses influence numerous physiological processes (expression of defense genes, synthesis of stress phytohormones, photosynthesis, respiration, phloem mass flow, ATP production, etc.) [7,8,9,10,11,12,13,14]. Increasing plant tolerance to the action of stressors is probably the main result of these physiological changes [13].

There are several ways of the participation of the H^+^-ATP-ases in electrical signaling in higher plants.

First, it is known that H^+^-ATP-ases support ion gradients across the plasma membrane [4,27,28]. Activation of ion channels (Ca^2+^, K^+^, and anion channels) seems to play important role in the generation of the local electrical responses, action potentials, variation potentials, and probably system potentials [6,7,8,9,10,11,12,13]. As a result, supporting the ion gradients is necessary for the generation of electrical responses under the actions of stressors.

Second, our theoretical analysis [29] shows that small decreasing H^+^-ATP-ase activities can decrease the magnitude of cooling which induces the generation of electrical response (i.e., decreasing temperature threshold is observed). The effect is caused by decreasing the difference between the initial membrane potential (which is dependent on H^+^-ATP-ase activities) and threshold membrane potential for the electrical response generation (which is dependent on parameters of ions channels participating in this generation) [29].

Third, changes in H^+^-ATP-ase activities can directly participate in the generation of electrical responses. Decreasing these activities play an important role in the generation of local electrical responses [16,17], action potentials [11,30], and variation potentials [7,8,9,10,11,12,13,14,31,32]; their increasing is probably the mechanism of forming system potentials [13,25,26].

Fourth, changes in H^+^-ATP-ase activities and extra and intracellular pH, which accompanies the generation of electrical responses, are probably an important mechanism of influence of electrical responses on physiological processes [10,13]. In particular, a fast photosynthetic inactivation, which can be induced by electrical responses (at least action potentials and variation potentials) [33,34,35,36,37,38,39,40,41], is caused by changes in H^+^-ATP-ase activities [32,42,43,44], inducing alkalization of apoplast and acidification of cytoplasm [42,43], and stroma and lumen of chloroplasts [45].

Thus, H^+^-ATP-ase activities are the important factor of regulation of electrical signaling in plants; their relations with electrical responses are actively investigated in experimental [16,30,31,32,44,46,47] and theoretical [29] works. Heterogeneity in spatial distributions of H^+^-ATP-ase activities can potentially influence electrical signaling in higher plants; i.e., it can be a new mechanism of regulation of the electrical response generation.

There are some points supporting the possibility. (i) Theoretical analysis of systems of electrically connected excitable cells (based of the FitzHugh–Nagumo model) shows that increasing stochastic spatial heterogeneity in thresholds of electrical responses can increase the sensitivity of the system to stimuli [48]. The effect is known as “diversity-induced resonance” (DIR) and is theoretically shown for biological systems (e.g., neuronal systems) [48,49]. Considering relations between H^+^-ATP-ase activities and the temperature threshold [29], it can be expected that the “DIR-like” effect can be observed in plants with stochastic spatial heterogeneity in the activities of H^+^-ATP-ases. (ii) There are potential reasons for the heterogeneity in H^+^-ATP-ase activities including the differences of the activities between different types of plant cells (see e.g., [27]) and the spatially heterogenous distribution of factors that modify these activities (e.g., light intensity which can activate H^+^-ATP-ase [50] and be dependent on the zone of a leaf or depth in the lamina). (iii) Previously, we showed that standard deviations of the magnitudes of the active components of the membrane potentials at rest, which could be used for the estimation of H^+^-ATP-ase activities, were about 20–30% for pea and wheat [32,51,52]. Moreover, our unpublished data shows that standard deviation between the active components of the membrane potentials in mesophyll cells of the same tobacco leaves are 43 ± 11%. (iv) Theoretical analysis of the generation of electrical responses with using single cell model shows that time-dependent stochastic fluctuations of H^+^-ATP-ase activities decrease the temperature thresholds for these responses [53]. The result theoretically supports that the generation of electrical responses in plants can be influenced by mechanisms liking to stochastic resonance [54,55,56], which is also related to changeability in thresholds, but this changeability is time-dependent and not directly related to the spatial localization of the cell.

Experimental analysis of the influence of heterogeneity in spatial distributions of H^+^-ATP-ase activities on the electrical response generation is difficult (especially for the stochastic or small-scale heterogeneity). In contrast, simulation is an effective tool for analysis of similar problems (e.g., DIR are mainly investigated on the basis of models of groups of electrically connected neuronal cells [48,49]). As a result, the aim of our work was to conduct on analysis of the influence of this heterogeneity on parameters of cooling-induced electrical responses by using a two-dimensional model of excitable plant cells with electrical connections (simple model of plant leaf).

## 2. Description of Model of Cooling-Induced Electrical Response in Two-Dimensional System of Plant Excitable Cells with Heterogeneity in H^+^-ATP-Ase Activities

**Figure 1:**
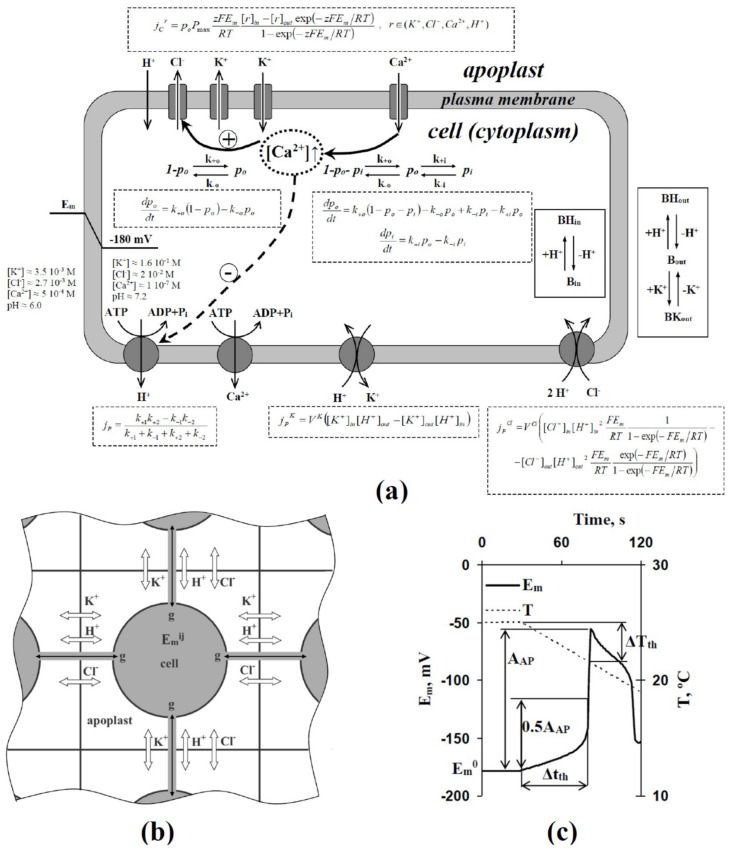
(**a**) Schema of the model of the generation of the cooling-induced electrical response (action potential in irritated zone, AP), (**b**) schema of interaction between neighboring cells, (**c**) and basic parameters of AP used in the current work. The model was based on our previous studies [11,29,53,57,58,59]; equations of the model are described in the File S1 (Supplementary Materials). The general model is composed of a two-dimensional group of cells (20 × 20 cells); each cell has its apoplast region. Each cell (excluding boundary cells) is connected with four neighboring cells. All cells are treated by cooling. E_m_ is a membrane potential; we analyze averaged E_m_ in the investigation (central 10 × 10 cells). E_m_^0^ is the value of E_m_ before the initiation of cooling; T is temperature; A_AP_ is the amplitude of AP; Δt_th_ is the duration of cooling, which is necessary for the induction of E_m_ changes equaling to 50% of A_AP_; and ΔT_th_ is magnitude of cooling, which is necessary for the induction of E_m_ changes equaling to 50% of A_AP_. E_m_^ij^ is the membrane potential in cell with coordinates i and j, and g is the electrical conductance between neighboring cells. *j_C_^r^* is the flux of ion r (Cl^−^, K^+^, Ca^2+^, or H^+^) through ion channels based on the Goldman–Hodgkin–Katz flux equation. Potential-dependent anion, inward, and outward K^+^ channels, Ca^2+^ channels, and H^+^ leakage (proton channels) are described. *p_o_* and *p_i_* are the probabilities of open and inactivated states of ion channels. *k_+o(+i)_* and *k_-o(-i)_* are velocity constants of transition from closed (open) to open (inactivated) states and vice versa. *P_max_* is the maximal permeability of the ion channel and z is the charge of the ion. F, R, and T are standard thermodynamic constants. *j_p_* is the flux of the ion through transport ATP-ases (H^+^-ATP-ase and Ca^2+^-ATP-ase described), which are described on the basis of the “two-state model”. *k_+1_*, *k_−1_*, *k_+2_*, and *k_−2_* are velocity constants for the transitions between states of the transport ATP-ases. Initial activities of H^+^-ATP-ases in different cells are modified by multiplication on stochastic variable ξ, which has normal distribution (ξ = 1 ± SD, where SD is the standard deviation). *j_p_^K^* is the flux through an H^+^-K^+^ symporter and *V^K^* is a parameter that is proportional to the rate of the transports of ions through the symporter. *j_p_^Cl^* is the flux through a 2H^+^-Cl^−^ antiporter and *V^Cl^* is a parameter that is proportional to the rate of the transports of ions through the antiporter. B_in_ and BH_in_ are free and H^+^-bound buffer molecules are in the cytoplasm. B_out_, BH_out_, and BK_out_ are free, H^+^-bound and K^+^-bound buffer molecules are in the apoplast. Cooling is imitated by the decrease of T with 4 °C min^−1^, 2 °C min^−1^, and 0.5 °C min^−1^ rates. Description of the modifications of transport enzymes activities are based on using Q_10_ = 3.

In our analysis, we used the mathematical model of the generation of the cooling-induced electrical response (action potential in irritated zone, AP), which was based on the two-dimensional system of electrically connected excitable plant cells (simple model of leaf) and included a description of the stochastic heterogeneity in initial H^+^-ATP-ase activities (Figure 1). The model was developed on the basis of a series of our early works [11,29,53,57,58,59] devoted to the simulation of electrical activity in higher plants. Equations of the model and parameters are described in detail in File S1 (Supplementary Materials).

Briefly, the description of electrogenesis of single cells included descriptions of passive ion fluxes through potential dependent Ca^2+^ channels, anion channels, inward and outward K^+^ channels, and H^+^ leakage (probably through H^+^ channels [60]), the primary active transport through H^+^- and Ca^2+^-ATP-ases, and the secondary active transport through H^+^-K^+^ symporters and 2H^+^-Cl^−^ antiporters (Figure 1a). The regulatory influence of the membrane potential on ion channels was described on the basis of transitions between closed, open, and inactivated states (for Ca^2+^ channels), or between closed and open states of channels (for anion, inward, and outward K^+^ channels). For anion channels and H^+^-ATP-ases, the regulation of these transporters by Ca^2+^ concentration in the cytoplasm was also described. The model described changes in cytoplasmic and apoplastic concentrations of Cl^−^, K^+^, and H^+^, and changes in cytoplasmic concentration of Ca^2+^, on the basis of their fluxes; buffer capacities of cytoplasm and apoplast were also described in the model. In accordance with our previous works [29,53,59], the membrane potential was described as a stationary function of ion fluxes through the plasma membrane. The simplification was not the influence of dynamics of potential changes for relatively slow plant AP [29]; however, it strongly accelerated the numerical analysis of the model.

The general model was composed of the two-dimensional system of cells (20 × 20 cells); each cell was electrically connected with four neighboring ones (excluding boundary cells) (Figure 1b). Each cell had its apoplast region. Diffusion ions between apoplasts of neighboring cells were described on the basis of the Fick’s law. All cells were treated by cooling. We analyzed the averaged membrane potential (central 10 × 10 cells) that was in accordance with the extracellular measurement of the potential.

In accordance with our previous work [53], stochastic heterogeneity in H^+^-ATP-ase activities was described by the multiplication of the flux through H^+^-ATP-ase on stochastic variable ξ, which had normal distribution (ξ = 1 ± SD, where SD is the standard deviation). It should be noted that ξ was stochastically calculated at the initiation of analysis only; after that, ξ was specific and constant for H^+^-ATP-ases in each simulated cell.

The model equations were numerically calculated by Euler’s method using the computer program Borland Delphi 7 which was developed for the solution of this task; the parameters and initial values of variables were shown in studies [29,53,57,58,59] and in File S1 (Supplementary Materials). The model included a stochastic variable; as a result, the Monte Carlo method was used for simulation (5 repetitions were used in the preliminary analysis and 25 repetitions were used in accurate analysis). Means, standard errors, and significances were calculated for investigated parameters.

In accordance with [53], we analyzed the membrane potential (E_m_) before cooling (E_m_^0^); amplitude of AP (A_AP_); duration of cooling, which was necessary for the induction of the membrane potential changes equaling to 50% of A_AP_ (Δt_th_); and magnitude of cooling, which was necessary for the induction of the membrane potential changes equaling to 50% of A_AP_ (ΔT_th_) (Figure 1c). It should be noted that we used this method of estimation of Δt_th_ and ΔT_th_ in accordance with [53] because the estimation of the inflection point time in the membrane potential under cooling can be more accurate for threshold estimation at constant parameters, while it can increase error in the system with stochastic parameters.

The parameters were analyzed at cooling with rates equaling to 4 °C min^−1^, 2 °C min^−1^, and 0.5 °C min^−1^. Only the first AP after initiation of the cooling was analyzed. The 4° C min^−1^ and 2 °C min^−1^ rates were investigated on the basis of using similar rates in earlier experimental investigations and simulations [29,30,53,57,58]; the rates were suitable for the experimental analysis of the cooling-induced electrical responses. The slow cooling (0.5 °C min^−1^), which was mainly used in our work, was selected as a tradeoff between slow rates of cooling in environmental conditions (not more than a few degrees per hour) and limitations of numerical simulations; e.g., simulation of 25 repetitions of 1 h dynamics of electrical activity (the single analyzed point) required about 7–8 h of real time.

Additionally, we analyzed standard deviations of the membrane potential before the cooling initiation (SD(E_m_^0^)), at 1 min after the cooling with 0.5 °C min^−1^ rate (SD(E_m_^1 min^)), and at 2 min after the cooling with 0.5 °C min^−1^ rate (SD(E_m_^2 min^)).

## 3. Results

### 3.1. Analysis of Influence of Stochastic Spatial Heterogeneity in H^+^-ATP-Ase Activities on Parameters of Cooling-Induced Electrical Responses

**Figure 2:**
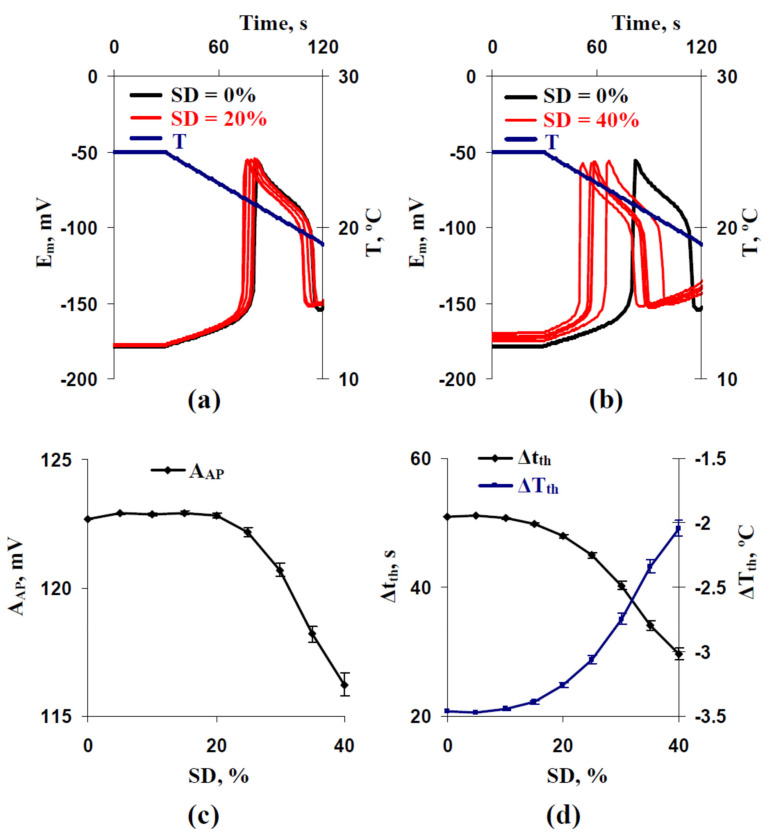
Examples of simulated changes in the membrane potential (E_m_) induced by a decrease of temperature (T) with a 4 °C min^−1^ rate at the standard deviation of initial H^+^-ATP-ase activity in cells (SD) equaling to (**a**) 20% and (**b**) 40%. (**c**) Dependences of the averaged amplitude of the first action potential (A_AP_) and (**d**) its time and temperature thresholds (Δt_th_ and ΔT_th_, respectively) on SD. Parameters of cooling-induced changes in E_m_ at SD = 0% were used as a control. Only five repetitions are shown in (**a**,**b**). Averaged parameters (A_AP_, Δt_th_, and ΔT_th_) shown in (**c**,**d**) were calculated on the basis of 25 repeated simulations.

The analysis of influence of the stochastic spatial heterogeneity in H^+^-ATP-ase activities on parameters of cooling-induced electrical responses (action potentials) was the first task of our work. It was shown (Figure 2a,b) that increasing standard deviation (SD) of these activities decreased durations of cooling with 4 °C min^−1^ rate, which was necessary for the induction of the first AP. The effect was stronger at SD = 40% than at SD = 20%. Figure 2c,d shows averaged values of AP parameters. It was shown that the time threshold (Δt_th_) and temperature threshold (ΔT_th_) decreased with increasing SD (Figure 2d); magnitudes of the changes were about 20 s and 1.5 °C, respectively. The amplitude of the first AP (A_AP_) was also dependent on SD (Figure 2c). A_AP_ was weakly changed at 0–20% SD and decreased at SDs, which were more than 20%.

**Figure 3:**
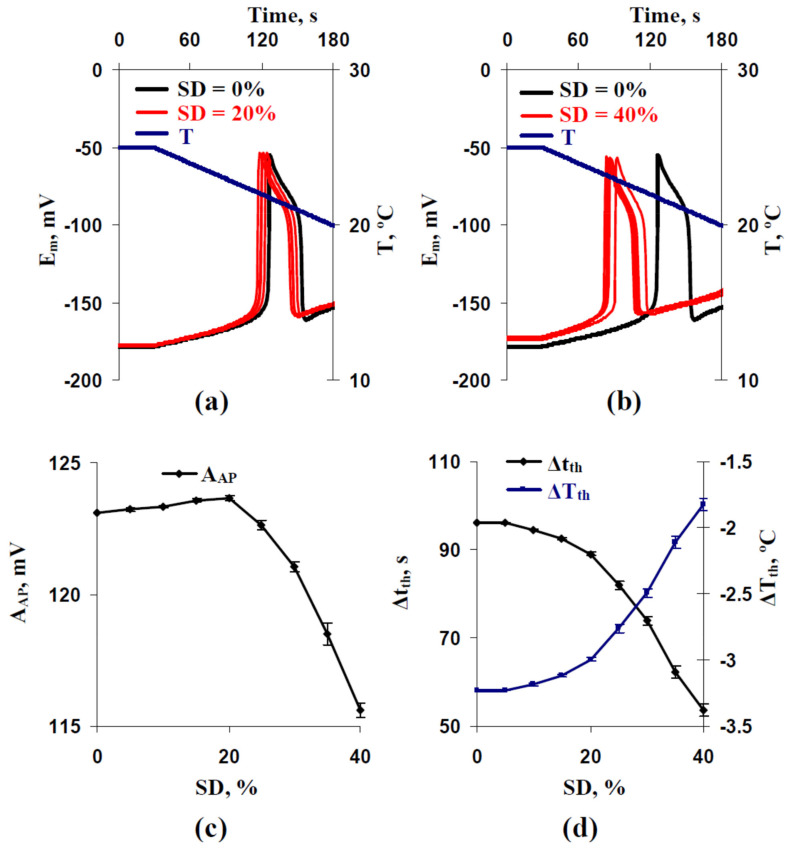
Examples of simulated changes in the membrane potential (E_m_) induced by a decrease of temperature (T) with a 2 °C min^−1^ rate at the standard deviation of initial H^+^-ATP-ase activity in cells (SD) equaling to (**a**) 20% and (**b**) 40%. (**c**) Dependences of the averaged amplitude of the first action potential (A_AP_) and (**d**) its time and temperature thresholds (Δt_th_ and ΔT_th_, respectively) on SD. Parameters of cooling-induced changes in E_m_ at SD = 0% were used as a control. Only five repetitions are shown in (**a**,**b**). Averaged parameters (A_AP_, Δt_th_, and ΔT_th_) shown in (**c**,**d**) were calculated on the basis of 25 repeated simulations.

**Figure 4:**
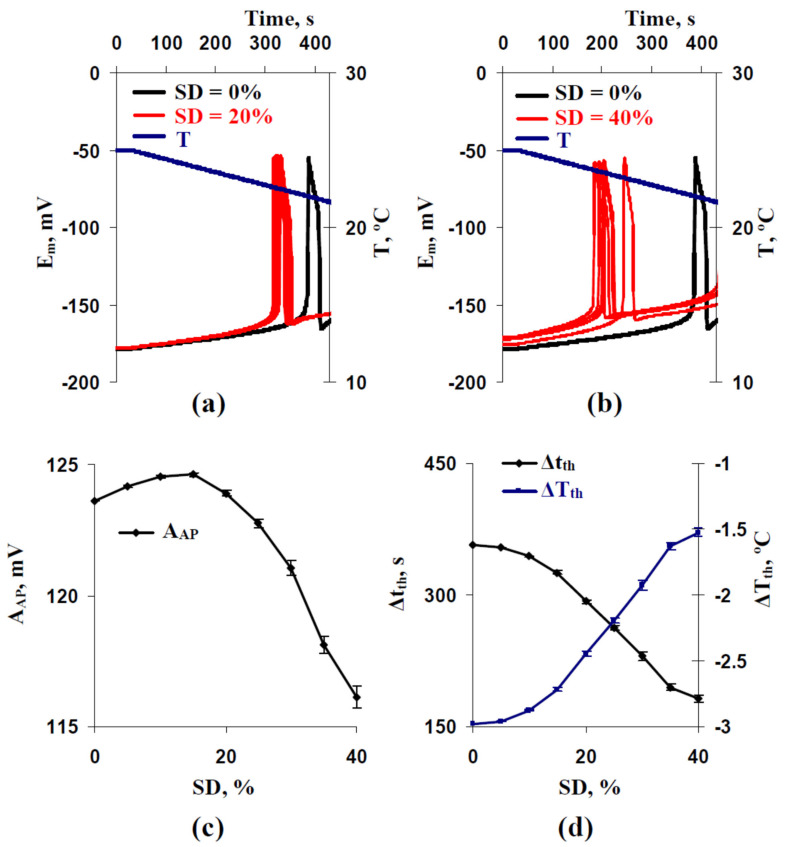
Examples of simulated changes in the membrane potential (E_m_) induced by a decrease of temperature (T) with a 0.5 °C min^−1^ rate at the standard deviation of initial H^+^-ATP-ase activity in cells (SD) equaling to (**a**) 20% and (**b**) 40%. (**c**) Dependences of the averaged amplitude of the first action potential (A_AP_) and (**d**) its time and temperature thresholds (Δt_th_ and ΔT_th_, respectively) on SD. Parameters of cooling-induced changes in E_m_ at SD = 0% were used as a control. Only five repetitions are shown in (**a**,**b**). Averaged parameters (A_AP_, Δt_th_, and ΔT_th_) shown in (**c**,**d**) were calculated on the basis of 25 repeated simulations.

Using other rates of the cooling, including 2 °C min^−1^ (Figure 3) and 0.5 °C min^−1^ (Figure 4), weakly influenced changes in the temperature threshold. Magnitudes of the decreased ΔT_th_ were also at about 1.5 °C (Figure 3d and Figure 4d). In contrast, decreases of Δt_th_ were strongly dependent on the rate of cooling and were about 40 s for 2 °C min^−1^ (Figure 3d) and about 175 s for 0.5 °C min^−1^ (Figure 4d). The last result was expected because ΔT_th_ was weakly changed at different cooling rates (e.g., −3.47 °C at the 4 °C min^−1^ rate (Figure 2d), −3.23 °C at the 2 °C min^−1^ rate (Figure 3d), and −2.98 °C at the 0.5 °C min^−1^ rate (Figure 4d) for control variants with SD = 0%). However, decreasing Δt_th_ from 357 s (SD = 0%) to 182 s (SD = 40%) at the 0.5 °C min^−1^ rate (Figure 4d) is potentially more important for plants than similarly decreasing Δt_th_ from 51 s (SD = 0%) to 30 s (SD = 40%) at the 4 °C min^−1^ rate (Figure 2d) because induction of fast physiological changes caused by electrical responses can be observed for 1–2 min (e.g., for adaptive photosynthetic changes [10,13]).

The dependence of A_AP_ on SD at the 0.5 °C min^−1^ rate (Figure 4c) differed from the similar dependence at the 4 °C min^−1^ rate (Figure 2c). At the 0.5 °C min^−1^ rate, increasing A_AP_ was observed with increasing SD from 0% to 15% and decreasing A_AP_ was observed with increasing SD from 15% to 40%. The magnitude of the increase was low (about 1 mV). At the 2 °C min^−1^ rate, the initial increasing A_AP_ was weak (Figure 3c); its magnitude was about 0.5 mV.

Thus, results of the first stage of analysis showed that the stochastic spatial heterogeneity in H^+^-ATP-ase activities influenced parameters of AP (especially time and temperature thresholds), induced by cooling with different rates. In the further analysis, we only used the 0.5 °C min^−1^ rate of cooling.

### 3.2. Two Potential Mechanisms of Influence of Stochastic Spatial Heterogeneity in H^+^-ATP-Ase Activities on Parameters of Cooling-Induced Electrical Responses

**Figure 5:**
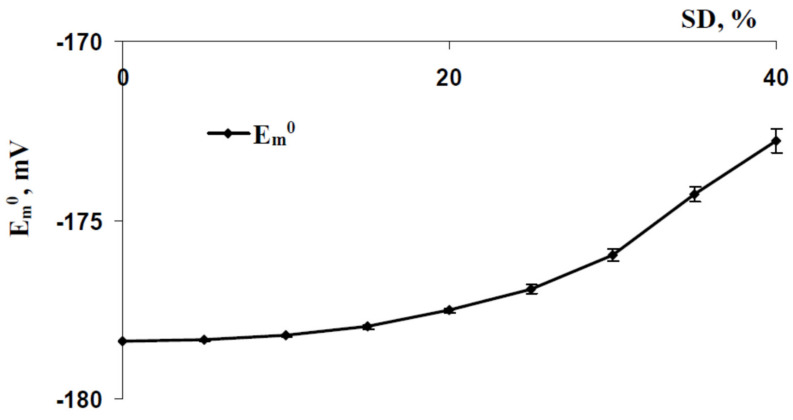
Dependence of the averaged simulated membrane potential at rest (E_m_^0^) on the standard deviation of initial H^+^-ATP-ase activity in cells (SD) (*n* = 25).

Figure 2b, Figure 3b and Figure 4b show that high SD (SD = 40%) induced weak a positive shift of the membrane potential at rest in comparison with this potential in the control (SD = 0%). Further analysis (Figure 5) supported that increasing SD induced increasing E_m_^0^ from about -178 mV (SD = 0%) to about −173 mV (SD = 40%).

**Figure 6:**
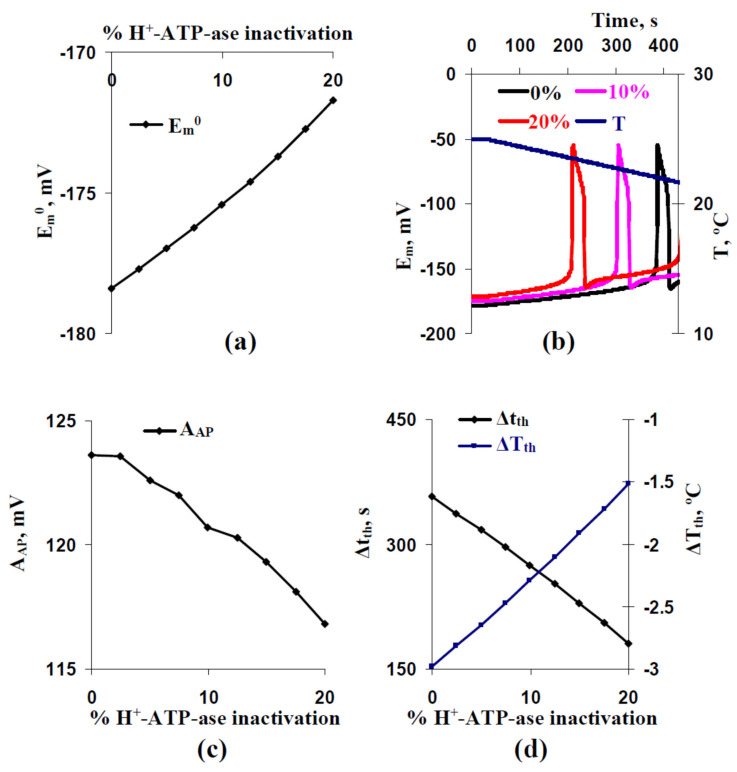
(**a**) Dependence of E_m_^0^ on percentage of H^+^-ATP-ase inactivation, (**b**) examples of simulated changes in E_m_ induced by the T decrease with a 0.5 °C min^−1^ rate at 0%, 10%, and 20% H^+^-ATP-ase inactivation, and (**c**) dependence of A_AP_ and (**d**) both Δt_th_ and ΔT_th_ on the percentage of H^+^-ATP-ase inactivation. The percentage of H^+^-ATP-ase inactivation shows a relative decrease of concentration of H^+^-ATP-ase in comparison to the control value (0% H^+^-ATP-ase inactivation).

Earlier, we theoretically demonstrated [29] that the weak positive shift of the membrane potential at rest, which was caused by the decrease of activities of H^+^-ATP-ases in the plasma membrane, could decrease the threshold of the cooling-induced AP generation. Current analysis, which was based on the modified model of the cooling-induced AP generation in the two-dimensional system of excitable plant cells, supported this result (Figure 6). It was shown that simultaneous decreasing of initial H^+^-ATP-ase activities in all cells of our model induced the decreasing of the membrane potential at rest (Figure 6a), AP amplitude (Figure 6c), and time and temperature thresholds of generation of the action potential (Figure 6d).

**Figure 7:**
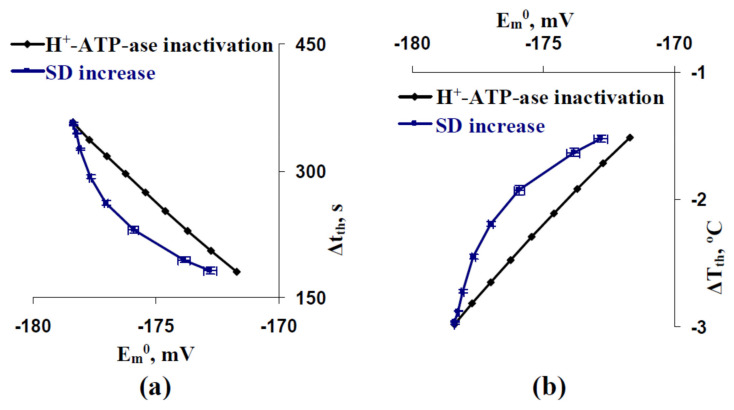
(**a**) Dependences of Δt_th_ and (**b**) ΔT_th_ on E_m_^0^ at different H^+^-ATP-ase inactivation (percentage) and different SD increase. Results from Figure 4, Figure 5 and Figure 6 were used.

The result showed that the positive shift of the membrane potential at rest could be mechanism of SD influence on thresholds of the cooling-induced AP generation. However, it was shown that dependences of Δt_th_ (Figure 7a) and ΔT_th_ (Figure 7b) on E_m_^0^ differed for simulations with different SD increase and simulations with different inactivation of H^+^-ATP-ases in all cells of the model. Decreasing time and temperature thresholds with increasing the membrane potential at rest was strongly linear at the different inactivations of H^+^-ATP-ases and non-linear at the different SD increases (the decreasing at weak positive shifts of E_m_^0^ was larger in this variant). In particular, E_m_^0^ = −176 mV was accompanied by Δt_th_ = 230 s and ΔT_th_ = −1.924 °C in the analysis with different SD increases and by Δt_th_ = 296 s and ΔT_th_ = −2.475 °C in the analysis with different inactivations of H^+^-ATP-ases. The results showed that decreasing E_m_^0^ was not the only mechanism of SD influence on time and temperature thresholds of the cooing-induced AP generation.

**Figure 8:**
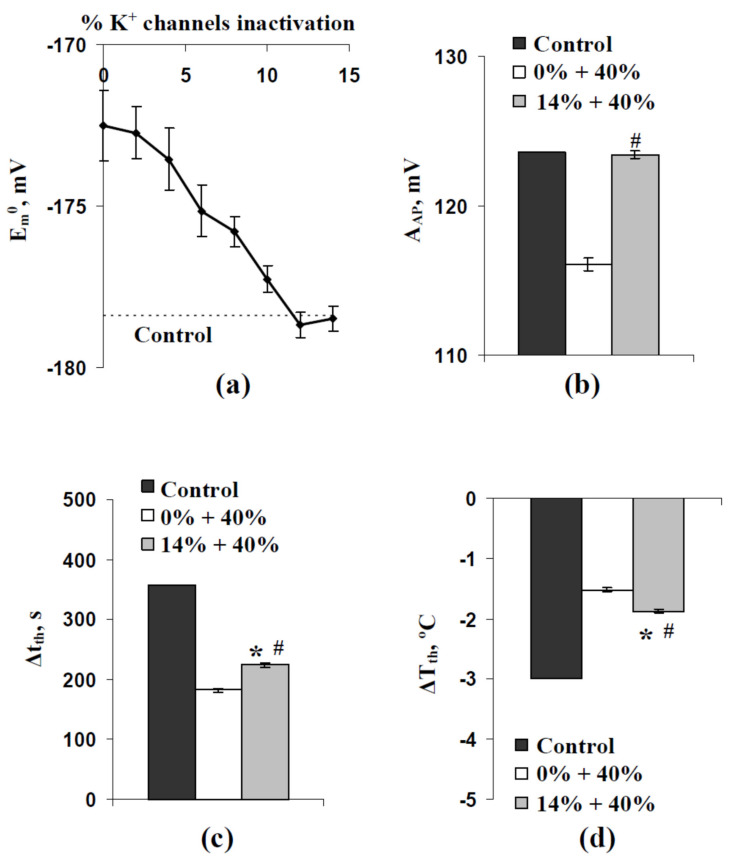
(**a**) Dependences of E_m_^0^ on percentage of inactivation of inward K^+^ channels at SD = 40% (*n* = 5) and (**b**) A_AP_, (**c**) Δt_th_, and (**d**) ΔT_th_ in the control (no inactivation of K^+^ channels, SD = 0%), the “0% + 40%” variant (no inactivation of K^+^ channels, SD = 40%), and the “14% + 40%” variant (14% of K^+^ channels inactivation; SD = 40%). *, the “14% + 40%” variant was significantly differed from the control. #, the “14% + 40%” variant was significantly differed from the “0% + 40%” variant. The rate of the T decrease was 0.5 °C min^−1^. Averaged parameters (A_AP_, Δt_th_, and ΔT_th_) shown in Figure 8b–d were calculated on the basis of 25 repeated simulations.

At rest, E_m_^0^ is mainly dependent on two parameters of ion transporters in the plasma membrane: activities of H^+^-ATP-ases and permeabilities of inward K^+^ channels. It can be expected that the weak decreasing of the membrane potential induced by increasing SD can be compensated by the decreasing permeabilities of these channels. Figure 8a shows that 12–14% of decreasing permeabilities of inward K^+^ channels fully eliminated changes in E_m_^0^ induced by increasing the standard deviation between initial activities of H^+^-ATP-ases in different cells of our model.

Further analysis showed that elimination of the decreasing E_m_^0^ eliminated the decreasing A_AP_ at high SD (SD = 40%) (Figure 8b). However, significant decreasing of Δt_th_ (Figure 8c) and ΔT_th_ (Figure 8d) were observed after this elimination. The result additionally showed that the SD-dependent decreasing of the membrane potential at rest was not the only mechanism of SD influence on time and temperature thresholds of the cooling-induced AP generation. Moreover, this decreasing was probable to induce the decreasing of the AP amplitude; however, increasing A_AP_ (see Figure 4c) was not caused by this mechanism.

### 3.3. Relations between Standard Deviations of the Membrane Potentials before Cooling-Induced AP Generation and Temperature Threshold of This Generation

Our results showed (Section 3.1 and Section 3.2) that increasing the stochastic heterogeneity between H^+^-ATP-ase activities in different plant cells, which were electrically connected, decreased the threshold of AP generation. It is known that the electrical activity of plants [61,62,63,64,65,66,67,68,69,70] or changes in plant reflectance caused by this activity [40,41,71,72] can be used for revealing actions of stressors and following physiological changes in plants. Considering the points discussed, we hypothesized that standard deviations of membrane potentials before the action potential generation could be related to the thresholds of generation of the cooling-induced APs.

**Figure 9:**
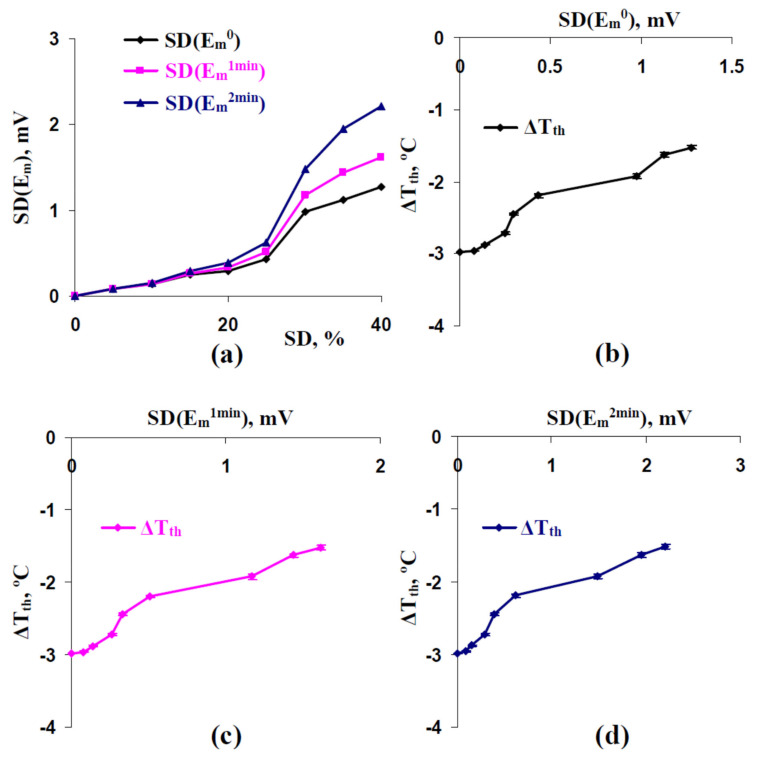
(**a**) Dependences of standard deviations of the membrane potential (SD(E_m_)) before the cooling initiation (SD(E_m_^0^)), at 1 min after initiations of the cooling with a 0.5 °C min^−1^ rate (SD(E_m_^1 min^)), and at 2 min after initiations of the cooling with a 0.5 °C min^−1^ rate (SD(E_m_^2 min^)) on standard deviation of initial H^+^-ATP-ase activity in cells (SD). (**b**) Dependence of temperature thresholds of the first action potential (ΔT_th_) on SD(E_m_^0^). (**c**) Dependences of ΔT_th_ on SD(E_m_^1 min^). (**d**) Dependences of ΔT_th_ on SD(E_m_^2 min^). SD(E_m_^0^), SD(E_m_^1 min^), and SD(E_m_^2 min^) were calculated on the basis of 25 repeated simulations. Averaged temperature thresholds (ΔT_th_) from Figure 4 were used.

Figure 9a shows that increasing SD was accompanied by increasing standard deviations of the membrane potential before the cooling initiation (SD(E_m_^0^)), at 1 min after the cooling with a 0.5 °C min^−1^ rate (SD(E_m_^1 min^)), and at 2 min after the cooling with a 0.5 °C min^−1^ rate (SD(E_m_^2 min^)). Durations of cooling equaling to 1 min and 2 min were selected because the generation of action potentials were absent for the time intervals at the 0.5 °C min^−1^ cooling rate. All dynamics were similar; however, the magnitude of increasing the standard deviation of the membrane potential was maximal at 2 min the cooling initiation. After that, it was shown that increasing SD(E_m_^0^) (Figure 9b), SD(E_m_^1 min^) (Figure 9c), and SD(E_m_^2 min^) (Figure 9d) were accompanied by increasing the temperature threshold of the AP generation.

It was additionally showed (Figure 9b–d) that dependences of temperature thresholds on SDs included two increases. The result could be explained by two mechanisms of influencing standard deviations in H^+^-ATP-ase activities on the thresholds: the first increase was caused by the mechanism, which was not related to the weak decreasing of the membrane potential at rest (probably the DIR [48,49]), and the second increase was caused by this weak decreasing.

Thus, the results showed that the stochastic heterogeneity between H^+^-ATP-ase activities in different plant cells (i.e., the spatial heterogeneity in H^+^-ATP-ase activities) could be estimated on the basis of standard deviations between averaged membrane potentials before the action potential generation. In turn, these standard deviations are related to thresholds of the cooling-induced action potential generation.

## 4. Discussion

Electrical responses play an important physiological role in plants [7,8,10,12,13,14]; they influence the expression of defense genes [73,74,75], synthesis of phytohormones [37,76,77], photosynthesis [33,34,35,36,37,38,39,40,41,42,43,45], phloem mass flow [78,79], respiration [32,80,81,82], and many other processes. Increasing plant tolerance to the action of stressors is probably the result of the physiological changes [13,17,52,83,84,85,86]. H^+^-ATP-ases are key electrogenic transporters for electrical responses in plants because their activities support ion gradients across the plasma membrane at rest [4,27,28] and influence thresholds for these responses [29]. Changes in these activities participate in the electrical response generation [7,8,9,10,11,12,13,14,16,17,25,26,30,31,32] and induction of physiological changes [32,44].

**Figure 10:**
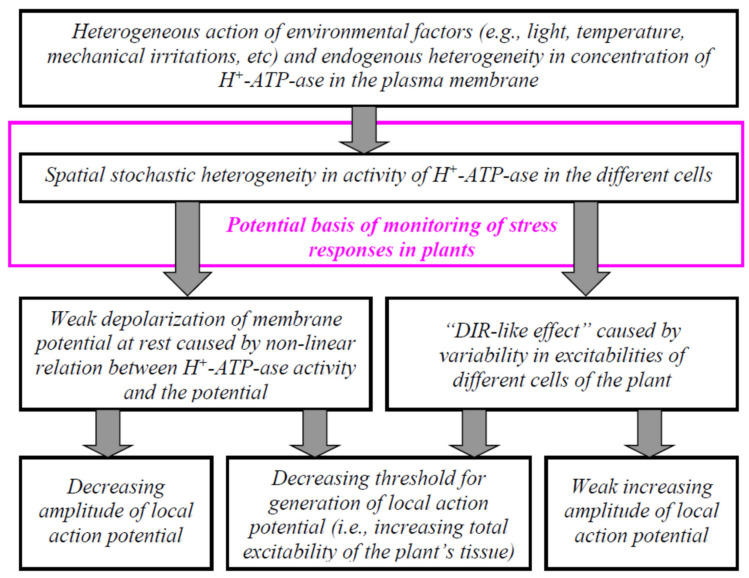
Hypothetical scheme of influencing stochastic spatial heterogeneity in the activity of H^+^-ATP-ase in different cells of the plant tissue on amplitudes and thresholds of local action potential in the zone of irritation. The scheme is based on our theoretical analysis of influencing the spatial heterogeneity on parameters of cooling-induced local action potentials. DIR is the diversity-induced resonance.

Considering the points discussed, changes in H^+^-ATP-ase activities can be the mechanism of regulation of plant electrical signaling and therefore can regulate plant responses to the actions of environmental factors. In the current work, we show (Figure 10) that the stochastic spatial heterogeneity of initial H^+^-ATP-ase activities, which is simulated by the stochastic heterogeneity between H^+^-ATP-ase activities in different plant cells of their system, influences time and temperature thresholds of generation of the cooling-induced action potentials (Figure 2, Figure 3 and Figure 4).

It is probable that there are two reasons for the revealed effects. First, we show that increasing the spatial heterogeneity of initial H^+^-ATP-ase activities decreases the membrane potential at rest (Figure 5). In turn, the positive shift of the membrane potential decreases the difference between the membrane potential at rest and the threshold membrane potential (which should be reached for AP generation). As a result, the magnitude of the temperature changes, which are necessary for AP induction, decreases; the duration of the cooling also decreases. The mechanism is in accordance with our early work [29] that theoretically showed decreasing temperature thresholds at weak decreasing H^+^-ATP-ase activities. The analysis of the direct influence of the decreasing H^+^-ATP-ase activities on thresholds in the current work (Figure 6) also supports this mechanism.

However, we had several points which showed that decreasing the membrane potential at rest is not the only mechanism of changes in thresholds at the increasing of the stochastic spatial heterogeneity of initial H^+^-ATP-ase activities. (i) Significant decreasing time and temperature thresholds can be observed at 5% and 10% increasing standard deviation of initial H^+^-ATP-ase activities (Figure 2d, Figure 3d and Figure 4d); however, changes in the membrane potential at rest are practically absent in these variants (Figure 5). (ii) Dependences of time and temperature thresholds on the membrane potential at rest are different for the simulation with different H^+^-ATP-ase inactivation and simulation with different increasing of the standard deviation between initial H^+^-ATP-ase activities (Figure 7). (iii) Artificial elimination of the positive shift of the membrane potential at rest does not fully eliminate the threshold changes (Figure 8). It can be proposed that the second mechanism of influence of the stochastic spatial heterogeneity in initial H^+^-ATP-ase activities on thresholds of AP generation is similar to diversity-induced resonance (DIR) [48,49].

DIR was theoretically shown by Tessone et al. [48] in a system of neuronal excitable elements (excitable cells) with the stochastic distribution of thresholds of the electrical response generation in these elements. The additional necessary condition for DIR is the global (each element is connected with all elements [48]) or local (each element is connected with nearest elements [87]) electrical connection between excitable elements in the system. In the case of DIR, moderate increasing dispersion of the thresholds in elements can increase sensitivity of the system to action of external stimuli [48]. The increase of the threshold dispersion associates to additionally formed elements with low and high thresholds of the electrical response generation in comparison with average thresholds in the system. External stimuli cause early excitation of elements with low thresholds of AP generation; after that, the combination of actions of the stimuli and APs in the low-threshold elements induces excitation of the average-threshold elements. Finally, the combination of actions of the stimuli and APs in the low-threshold and average-threshold elements induces excitation of the high-threshold elements.

DIR is mostly shown on the basis of a simple model of action potentials (e.g., the FitzHugh–Nagumo model [48,87,88]) but more complex models are also used in a few studies (e.g., the Hodgkin–Huxley-type models [89]). Our results show that a similar effect can be revealed on the basis of a complex model of electrical responses in plants, which was developed in series of our previous works [11,29,53,57,58]. Increasing the stochastic spatial heterogeneity of initial H^+^-ATP-ase activities is probable to modify thresholds of AP generation in individual cells because weak changes in these activities strongly influence AP thresholds [29]. Dispersion of the individual thresholds causes DIR-like effects and decreases the total threshold of the simulated system of plant cells for electrical responses (particularly cooling-induced action potentials in the irritated zone).

It should be also noted that increasing the spatial heterogeneity of initial H^+^-ATP-ase activities influences amplitudes of the cooling-induced action potentials (Figure 2c, Figure 3c and Figure 4c). The main effect is decreasing the amplitudes at the high heterogeneity, which is caused by decreasing the membrane potential at rest (Figure 6c and Figure 8b). Weak increasing A_hv_ is probably related to other mechanisms which require future investigation; however, the low magnitude of the effect (up to 1 mV) limits its potential significance for plants.

Considering the strong relations of electrical responses and physiological processes [7,8,10,12,13,14], we suppose that the revealed decrease of AP thresholds under increasing the spatial heterogeneity of initial H^+^-ATP-ase activities can be potentially important for plant adaptation to action of environmental stressors. It can be expected that the effect is more important under actions of stressors with a low rate of intensity increase (e.g., decreasing temperature with low rate). The last point is explained by time intervals, which are necessary for the induction of physiological changes by electrical responses (e.g., induction of photosynthetic [36,42,43] and respiratory [80,81,82] changes by electrical signals requires about 1–2 min; decreasing phloem mass flow after propagation of these signals requires several minutes [79], etc.).

Increasing plant tolerance to stressors is the result of the influence of electrical responses on physiological processes [10,12,13]; it indicates that the acceleration of the response generation can contribute to plant survival under actions of stressors (e.g., cooling). Our results show that increasing the stochastic spatial heterogeneity in H^+^-ATP-ases activities can be the mechanism of increasing plant tolerance through decreasing lag time between the initiation of the stressor action and the electrical response generation. It can be supposed that this positive effect can be rather observed under the action of slow-acting stressors (adaptive changes have time to form in this case). As a whole, the results are in a good accordance with the concept regarding the “constructive role” of stochastic processes in biosystems [90].

It is known that the electrical activity of plants [61,62,63,64,65,66,67,68,69,70] or changes in plant reflectance caused by this activity [40,41,71,72] can be used for revealing actions of stressors and following physiological changes in plants. Considering that point, our results additionally show (Figure 9) that standard deviation between averaged membrane potentials before the first AP induction (the membrane potentials at rest or the potentials under weak actions of stressors) can be potentially used for the estimation of plant electrical responses on actions of stressors and probably their adaptations to these actions. Thus, it can be speculated that measuring surface electrical potentials in different parts of plants and calculating their standard deviation (or measuring plant reflectance parameters, which are related to electrical responses [40,41,71,72]) can be used as an estimator (and perhaps a predictor) of plant stress responses.

Finally, some perspectives of the development of our model should be discussed. First, influencing other types of spatial heterogeneity on the generation of electrical signals can be theoretically analyzed (e.g., large-scale spatial heterogeneity in H^+^-ATP-ase activities or stochastic spatial heterogeneity in permeabilities of Ca^2+^ channels, conductance of plasmodesmata, and other processes, which are crucial for the AP generation [6,7,8,9,10,11,12,13,14]). The analyses can be performed on the basis of our model after its moderate modifications. Second, the spatial heterogeneities can potentially influence the propagation of action potentials because, e.g., changes in H^+^-ATP-ase activities and intercellular conductance can strongly influence AP propagation [29]. Third, the generation and propagation of electrical responses in plants (especially variation potentials) can be related to numerous signal processes including hydraulic waves, Ca^2+^ waves, reactive oxygen species (ROS) waves, changes in pH, stimulation of production of stress phytohormones, etc. [9,10,11,12,13,14,22,75,91,92,93,94,95,96,97]. The processes can be strongly interacted, induce numerous physiological changes, and increase plant tolerance to stressors [10,11,12,13,98,99]. It is very probable that “DIR-like” mechanisms can also influence these signaling processes; however, the analysis of the problem requires further development of their specific models.

## 5. Conclusions

H^+^-ATP-ases are key transporters for electrical signaling in plants. In the current study, we theoretically showed that increasing the stochastic spatial heterogeneity of initial H^+^-ATP-ase activities decreased thresholds for cooling-induced action potentials. Two potential mechanisms of this decrease were revealed. The first mechanism was based on decreasing the membrane potential at rest, which was accompanied by increasing the spatial heterogeneity. The second mechanism was not based on changes in the membrane potential at rest. It was probable that this mechanism was like diversity-induced resonance, which was mainly shown for electrically connected neuronal cells. Additionally, we showed that the standard deviation of membrane potentials before the induction of action potentials (e.g., the membrane potentials at rest) could be used for the estimation of thresholds of cooling-induced plant electrical responses.

## Data Availability

The data presented in this study are available upon request from the corresponding author.

## Supplementary Materials

The following is available online at https://www.mdpi.com/article/10.3390/ijms22158254/s1, File S1: Description of equations of the model of the cooling-induced local action potential and used parameters.
