# Supplementary material for: Stochastic Spatial Heterogeneity in Activities of H+-ATP-Ases in Electrically Connected Plant Cells Decreases Threshold for Cooling-Induced Electrical Responses
Source: Int J Mol Sci. 2021 Jul 31;22(15):8254. doi: 10.3390/ijms22158254 (PMC8348073; doi:10.3390/ijms22158254)
Supplement: Supplementary file 1 [file ijms-22-08254-s001.zip › ijms-1309615-supplementary.pdf]

Supplementary Materials

Description of equations of the model of the cooling-induced local action potential and used parameters

1. General structure and equations of the model

1.1. General structure of the model

The general structure includes the two-dimensional group of cells (20 × 20 cells). Each cell has its region of the apoplast, which is described as single volume. Each cell (excluding boundary cells) is connected with 4 neighboring cells. There are electrical connections between intracellular spaces of the cells through plasmodesmata and connections between the apoplast volumes through diffusion fluxes.

Generation of electrical responses in each cell is based on our earlier model of action potential generation in higher plants. The model includes description of active and passive fluxes of ions, their regulations, changes in concentrations of ions, buffers in cytoplasm and apoplast, etc.

All cells are treated by cooling. Central region (10 × 10 cells) is analyzed in the model. Membrane potentials of the cells are averaged during simulation.

1.2. General equations of the model

We use stationary description of the membrane potential (E_m) because generation of local action potentials (APs) in plant is relatively slow processes and differences between stationary E_m and E_m, calculated on basis of differential equation, are very small. E_m in specific cell *ij* (E_m^{ij}) is described by equation (S1):

$$E_m^{ij} = \frac{gE_{tot}^{ij} + gE_m^{i-1j} + gE_m^{i+1j} + gE_m^{ij-1} + gE_m^{ij+1}}{g_{tot}^{ij} + 4g} \qquad \text{(S1)}$$

where E_m^{i-1j}, E_m^{i+1j}, E_m^{ij-1}, and E_m^{ij+1} are the membrane potentials of 4 neighboring cells; g is conductance between cells (1000 S m^{-2}); gE_{tot} and g_{tot} are additional variables, which are calculated by Equations (S2) and (S3) for each cell ij. If one or two neighboring cells are absent (boundary cells) then their E_m and g are assumed to equaling to zero.

$$gE_{tot} = g_C^{A}E_{Cl} + g_P^{Cl}E_P^{Cl} + g_C^{Ca}E_{Ca} + g_P^{Ca}E_P^{Ca} + \left(g_{inC}^{K} + g_{outC}^{K}\right)E_K + g_C^{H}E_H + g_P^{H}E_P \qquad \text{(S2)}$$

$$g_{tot} = g_C^{A} + g_P^{Cl} + g_C^{Ca} + g_P^{Ca} + g_{inC}^{K} + g_{outC}^{K} + g_C^{H} + g_P^{H} \qquad \text{(S3)}$$

where g_C^A, g_P^{Cl}, g_C^{Ca}, g_P^{Ca}, g_{inC}^K, g_{outC}^K, g_C^H, and g_P^H are conductance for the anion channel, $2H^+$-Cl^- antiporter, calcium channel, Ca^{2+}-ATP-ase, inward and outward K^+ channels, H^+ leakage (proton channels), and H^+-ATP-ase, respectively. E_{Cl}, E_{Ca}, E_K, and E_H are the Nernst potentials for Cl^-, Ca^{2+}, K^+, and H^+, respectively. E_P^{Cl}, E_P^{Ca}, and E_P^H are electromotive forces caused by $2H^+$-Cl^- antiporter, calcium channel, Ca^{2+}-ATP-ase, and H^+-ATP-ase, respectively.

Equations (S4)-(S11) are used for calculation of g_C^A, g_P^{Cl}, g_C^{Ca}, g_P^{Ca}, g_{inC}^K, g_{outC}^K, g_C^H, and g_P^H.

$$g_C^{A} = \frac{i_C^{A}}{E_m - E_{Cl}} \qquad \text{(S4)}$$

$$g_P^{Cl} = \frac{i_P^{Cl}}{E_m - E_P^{Cl}} \tag{S5}$$

$$g_C^{Ca} = \frac{i_C^{Ca}}{E_m - E_{Ca}} \tag{S6}$$

$$g_P^{Ca} = \frac{i_P^{Ca}}{E_m - E_P^{Ca}} \tag{S7}$$

$$g_{inC}^{K} = \frac{i_{inC}^{K}}{E_m - E_K} \tag{S8}$$

$$g_{outC}^{K} = \frac{i_{outC}^{K}}{E_m - E_K} \tag{S9}$$

$$g_C^{H} = \frac{i_C^{H}}{E_m - E_H} \tag{S10}$$

$$g_P^{H} = \frac{i_P^{H}}{E_m - E_P^{H}} \tag{S11}$$

where i_C^A, i_P^{Cl}, i_C^{Ca}, i_P^{Ca}, i_{inC}^K, i_{outC}^K, i_C^H, and i_P^H are current densities for the anion channel, $2H^+$-Cl^- antiporter, calcium channel, Ca^{2+}-ATP-ase, inward and outward K^+ channels, H^+ leakage (proton channels), and H^+-ATP-ase, respectively. The current densities are functions of ion fluxes which are described below.

2. Descriptions of ion fluxes and their regulations

2.1. Potential-dependent Ca^{2+} channels

The Goldman–Hodgkin–Katz flux equation is used for description of the Ca^{2+} flux through the potential-dependent Ca^{2+} channels (j_C^{Ca}):

$$j_C^{Ca} = p_o P_{\max} \frac{zFE_m}{RT} \frac{[Ca^{2+}]_{in} - [Ca^{2+}]_{out} \exp(-zFE_m/RT)}{1 - \exp(-zFE_m/RT)} \tag{S12}$$

where p_o and P_{max} (1.5×10^{-9} m s^{-1}) are probability of open state and maximal permeability of the channel, respectively. z (+2) is charge of Ca^{2+}. F (96 500 C mol^{-1}), R (8.31 J K^{-1}), and T are standard thermodynamic parameters. $[Ca^{2+}]_{out}$ and $[Ca^{2+}]_{in}$ are concentrations of calcium ions in apoplast and cytoplasm, respectively. It is assumed that $[Ca^{2+}]_{out}$ is constant (0.0005 M)

Differential equations (S13) are used for description of p_o:

$$\frac{dp_o}{dt} = k_{+o}(1 - p_o - p_i) - k_{-o}p_o + k_{-i}p_i - k_{+i}p_o$$
$$\frac{dp_i}{dt} = k_{+i}p_o - k_{-i}p_i \tag{S13}$$

where p_i is probability of inactivated state of the ion channel; $k_{+o(+i)}$ and $k_{-o(-i)}$ are velocity constants of transition from closed (open) to open (inactivated) states and vice versa.

k_{+o}, k_{+i}, k_{-o}, and k_{-i} are described by equations (S14):

$$k_{+o} = k_o \exp\left(\frac{E_m C^o}{2RT}\right)$$

$$k_{-o} = k_o \exp\left(\frac{2C^o E^o - C^o E_m}{2RT}\right) \quad \text{(S14)}$$

$$k_{+i} = k_i \exp\left(\frac{E_m C^i}{2RT}\right)$$

$$k_{-i} = k_i \exp\left(\frac{2C^i E^i - C^i E_m}{2RT}\right)$$

where C^o (111.437 J mV^{-1}) and C^i (123.819 J mV^{-1}) are the constants, which are dependent on portion of the membrane potential acting the gating mechanisms and on charges of the mechanisms; E^o (-90 mV) and E^i (-180.6 mV) are difference between potential energies (in millivolts) of the closed state and the open one and of the open state and the inactivated one, respectively, at E_m = 0 mV; k_o (353 s^{-1}) and k_i (0.6 s^{-1}) are velocity constants of transitions between the closed and open states and between the open and inactivated states at E_m=0, E^o=0, and E^i=0.

Equation (S15) is used for calculation of $i_C{}^{Ca}$:

$$i_C{}^{Ca} = zFj_C{}^{Ca} \quad \text{(S15)}$$

The Nernst equation (S16) is used for calculation of E_{Ca}:

$$E_{Ca} = \frac{RT}{zF} \ln\left(\frac{[Ca^{2+}]_{out}}{[Ca^{2+}]_{in}}\right) \quad \text{(S15)}$$

2.3. Potential-dependent inward K^+ channels

The Goldman–Hodgkin–Katz flux equation is used for description of the K^+ flux through the potential-dependent inward K^+ channels ($j_{inC}{}^K$):

$$j_{inC}{}^K = p_o(1-C)P_{\max} \frac{zFE_m}{RT} \frac{[K^+]_{in} - [K^+]_{out} \exp(-zFE_m/RT)}{1-\exp(-zFE_m/RT)} \quad \text{(S16)}$$

where p_o and P_{max} (2.9×10^{-8} m s^{-1}) are probability of open state and maximal permeability of the channel, respectively; z (+1) is charge of K^+. F (96 500 C mol^{-1}), R (8.31 J K^{-1}), and T are standard thermodynamic parameters; $[K^+]_{out}$ and $[K^+]_{in}$ are concentrations of potassium ions in apoplast and cytoplasm, respectively; C is parameter, which can be used for correction of maximal permeability of the potential-dependent inward K^+ channels (by default, C=0).

Differential equation (S17) is used for description of p_o:

$$\frac{dp_o}{dt} = k_{+o}(1-p_o) - k_{-o}p_o \quad \text{(S17)}$$

where k_{+o} and k_{-o} are velocity constants of transition from closed to open states and vice versa.

Stationary solution of the equation (S17) is used for simulation because times of activation and inactivation of the channels are strongly lower than durations of local action potentials in higher plants:

$$p_o = \frac{1}{1 + k_{-o}/k_{+o}} \tag{S18}$$

k_{-o}/k_{+o} is described by equation (S19):

$$k_{-o}/k_{+o} = \exp\left(\frac{C^o E^0 - E_m C^o}{RT}\right) \tag{S19}$$

where C^o (106.4843 J mV^{-1}) is the constants, which is dependent on portion of the membrane potential acting the gating mechanisms and on charges of the mechanisms; E^o (-190 mV) is the difference between potential energies (in millivolts) of the closed state and the open one at E_m = 0 mV.

Equation (S20) is used for calculation of $i_{inC}{}^K$:

$$i_{inC}{}^K = zFj_{inC}{}^K \tag{S20}$$

The Nernst equation (S21) is used for calculation of E_K:

$$E_K = \frac{RT}{zF}\ln\left(\frac{[K^+]_{out}}{[K^+]_{in}}\right) \tag{S21}$$

2.4. Potential-dependent outward K^+ channels

The Goldman–Hodgkin–Katz flux equation is used for description of the K^+ flux through the potential-dependent outward K^+ channels ($j_{outC}{}^K$):

$$j_{outC}{}^K = p_o P_{max} \frac{zFE_m}{RT} \frac{[K^+]_{in} - [K^+]_{out} \exp(-zFE_m/RT)}{1 - \exp(-zFE_m/RT)} \tag{S22}$$

where p_o and P_{max} (2.9×10^{-8} m s^{-1}) are probability of open state and maximal permeability of the channel, respectively; z (+1) is charge of K^+. F (96 500 C mol^{-1}), R (8.31 J K^{-1}), and T are standard thermodynamic parameters; $[K^+]_{out}$ and $[K^+]_{in}$ are concentrations of potassium ions in apoplast and cytoplasm, respectively.

Differential equation (S17) is used for description of p_o:

$$\frac{dp_o}{dt} = k_{+o}(1 - p_o) - k_{-o}p_o \tag{S23}$$

where k_{+o} and k_{-o} are velocity constants of transition from closed to open states and vice versa.

Stationary solution of the equation (S17) is used for simulation because times of activation and inactivation of the channels are strongly lower than durations of local action potentials in higher plants:

$$p_o = \frac{1}{1 + k_{-o}/k_{+o}} \tag{S24}$$

k_{-o}/k_{+o} is described by equation (S19):

$$k_{-o}/k_{+o} = \exp\left(\frac{C^oE^0 - E_mC^o}{RT}\right) \quad \text{(S25)}$$

where C^o (108.9607 J mV^{-1}) is the constants, which is dependent on portion of the membrane potential acting the gating mechanisms and on charges of the mechanisms; E^o (-65 mV) is the difference between potential energies (in millivolts) of the closed state and the open one at E_m = 0 mV.

Equation (S20) is used for calculation of i_{outC}^K:

$$i_{outC}^{\ \ K} = zFj_{outC}^{\ \ K} \quad \text{(S26)}$$

2.5. Potential- and Ca^{2+}-dependent anion channels

The Goldman–Hodgkin–Katz flux equation is used for description of the Cl^- flux through the potential- and Ca^{2+}-dependent anion channels (j_C^A):

$$j_C^{\ A} = p_oA^{Ca}P_{\max}\frac{zFE_m}{RT}\frac{[Cl^-]_{in} - [Cl^-]_{out}\exp(-zFE_m/RT)}{1-\exp(-zFE_m/RT)} \quad \text{(S27)}$$

where p_o and P_{max} (2.45×10^{-8} m s^{-1}) are probability of open state and maximal permeability of the channel, respectively; z (-1) is charge of Cl^-. F (96 500 C mol^{-1}), R (8.31 J K^{-1}), and T are standard thermodynamic parameters; $[Cl^-]_{out}$ and $[Cl^-]_{in}$ are concentrations of chlorine ions in apoplast and cytoplasm, respectively; A^{Ca} is variable describing activation of the channels by Ca^{2+} ions.

Differential equation (S28) is used for description of p_o:

$$\frac{dp_o}{dt} = k_{+o}(1-p_o) - k_{-o}p_o \quad \text{(S28)}$$

where k_{+o} and k_{-o} are velocity constants of transition from closed to open states and vice versa.

Stationary solution of the equation (S28) is used for simulation because times of activation and inactivation of the channels are strongly lower than durations of local action potentials in higher plants:

$$p_o = \frac{1}{1+k_{-o}/k_{+o}} \quad \text{(S29)}$$

k_{-o}/k_{+o} is described by equation (S30):

$$k_{-o}/k_{+o} = \exp\left(\frac{C^oE^0 - E_mC^o}{RT}\right) \quad \text{(S30)}$$

where C^o (247.638 J mV^{-1}) is the constants, which is dependent on portion of the membrane potential acting the gating mechanisms and on charges of the mechanisms; E^o (-120 mV) is the difference between potential energies (in millivolts) of the closed state and the open one at E_m = 0 mV.

A^{Ca} is described by equation (S31):

$$A^{Ca} = \frac{[Ca^{2+}]_{in}{}^2}{[Ca^{2+}]_{in}{}^2 + K_d{}^2} \left(\frac{\left(2.1\times10^{-6}\right)^2}{\left(2.1\times10^{-6}\right)^2 + K_d{}^2} \right)^{-1} \tag{S31}$$

where K_d (8×10^{-6} M) is dissociation constant between Ca^{2+} and the anion channel. 2.1×10^{-6} M is assumed as concentration of Ca^{2+} in cytoplasm at peak of action potential in higher plants

Equation (S32) is used for calculation of $i_C{}^A$:

$$i_C{}^A = zFj_C{}^A \tag{S32}$$

The Nernst equation (S33) is used for calculation of E_{Cl}:

$$E_{Cl} = \frac{RT}{zF}\ln\left(\frac{[Cl^-]_{out}}{[Cl^-]_{in}}\right) \tag{S33}$$

2.6. H^+ leakage (H^+ channels)

The Goldman–Hodgkin–Katz flux equation is used for description of the H^+ leakage (probably, through the H^+ channels) ($j_C{}^H$):

$$j_C{}^H = P\frac{zFE_m}{RT}\frac{[H^+]_{in} - [H^+]_{out}\exp(-zFE_m/RT)}{1-\exp(-zFE_m/RT)} \tag{S34}$$

where P (1×10^{-5} m s^{-1}) is permeability of the channel; regulation of H^+ leakage is not considered; z (+1) is charge of H^+; F (96 500 C mol^{-1}), R (8.31 J K^{-1}), and T are standard thermodynamic parameters; $[H^+]_{out}$ and $[H^+]_{in}$ are concentrations of protons in apoplast and cytoplasm, respectively.

Equation (S32) is used for calculation of $i_C{}^H$:

$$i_C{}^H = zFj_C{}^H \tag{S35}$$

The Nernst equation (S33) is used for calculation of E_H:

$$E_H = \frac{RT}{zF}\ln\left(\frac{[H^+]_{out}}{[H^+]_{in}}\right) \tag{S36}$$

2.7. H^+-ATP-ase and its regulation

The "two-state model" is used for description of H^+-ATP-ase in the plasma membrane. Equation (S37) describes active H^+ flux through the transporter ($j_P{}^H$):

$$j_P{}^H = \xi(1+C)I^{Ca}I^T\frac{k_{+1}k_{+2} - k_{-1}k_{-2}}{k_{+1}+k_{+2}+k_{-1}+k_{-2}} \tag{S37}$$

where k_{+1}, k_{-1}, k_{+2}, and k_{-2} are velocity constants for transitions between states of the H^+-ATP-ase; ξ =1±SD is stochastic variable with the normal distribution (SD is standard deviation), which simulates heterogeneity in activities of the H^+-ATP-ases in different plant cells; C is parameter, which can be used for correction of maximal activity of the H^+-ATP-ases (by default, C=0); I^{Ca} is variable describing inactivation of the H^+-ATP-ases by Ca^{2+} ions; I^T is variable describing regulation of activities of the H^+-ATP-ases by temperature (T). It should be noted that ξ are generated in each cell before initiation of simulation; after that, ξ is not changed.

Equations (S38) are used for calculation k_{+1}, k_{-1}, k_{+2}, and k_{-2}:

$$k_{+1} = k_1 [H^+]_{in}$$

$$k_{-1} = k_1 \exp(\Delta G_{ATP}/RT) \quad \text{(S38)}$$

$$k_{+2} = \frac{k_2 F E_m}{RT} \frac{1}{1-\exp(-FE_m/RT)}$$

$$k_{-2} = \frac{k_2 [H^+]_{out} F E_m}{RT} \frac{\exp(-FE_m/RT)}{1-\exp(-FE_m/RT)}$$

where $[H^+]_{out}$ and $[H^+]_{in}$ are concentrations of protons in apoplast and cytoplasm, respectively; k_1 (4.5×10^{-2} s^{-1}) and k_2 (2.58×10^{-5} s^{-1}) are velocity constants at E_m=0 mV and $[H^+]_{out} = [H^+]_{in} = 1$ M; ΔG_{ATP}=-50 000 J mol^{-1} is the energy of the ATP hydrolysis; F (96 500 C mol^{-1}), R (8.31 J K^{-1}), and T are standard thermodynamic parameters.

I^{Ca} is described by equation (S39):

$$I^{Ca} = \frac{K_d^{\ 2}}{[Ca^{2+}]_{in}^{\ 2} + K_d^{\ 2}} \left(\frac{K_d^{\ 2}}{(10^{-7})^2 + K_d^{\ 2}} \right)^{-1} \quad \text{(S39)}$$

where K_d (4×10^{-7} M) is dissociation constant between Ca^{2+} and the anion channel. 10^{-7} M is assumed as concentration of Ca^{2+} in cytoplasm at rest in higher plants.

I^T is described by equation (S40):

$$I^T = Q_{10}^{\frac{T_0 - T}{10}} \quad \text{(S40)}$$

where Q_{10} (3) is the temperature coefficient of the H^+-ATP-ase, T_0 (298 K) is initial temperature.

Equation (S41) is used for calculation of i_P^H:

$$i_P^{\ H} = zFj_P^{\ H} \quad \text{(S41)}$$

where z (+1) is charge, which is transferred per 1 enzyme turnover.

Equation (S42) is used for calculation of E_P^H:

$$E_P^{\ H} = \frac{\Delta G_{ATP}}{F} + \frac{RT}{F} \ln\left(\frac{[H^+]_{out}}{[H^+]_{in}} \right) \quad \text{(S42)}$$

2.8. Ca^+-ATP-ase

The "two-state model" is used for description of Ca^{2+}-ATP-ase in the plasma membrane. Equation (S43) describes active Ca^{2+} flux through the transporter (j_P^{Ca}):

$$j_P^{\ Ca} = I^T \frac{k_{+1}k_{+2} - k_{-1}k_{-2}}{k_{+1} + k_{+2} + k_{-1} + k_{-2}} \quad \text{(S43)}$$

where k_{+1}, k_{-1}, k_{+2}, and k_{-2} are velocity constants for transitions between states of the Ca^{2+}-ATP-ase; I^T is variable describing regulation of activities of the Ca^{2+}-ATP-ases by temperature (T).

We include antiport between Ca^{2+} and H^+ in the transport cycle of Ca^{2+}-ATP-ase. Equations (S44) are used for calculation k_{+1}, k_{-1}, k_{+2}, and k_{-2}:

$$k_{+1} = k_1 [Ca^{2+}]_{in} [H^+]_{out}$$

$$k_{-1} = k_1 \exp(\Delta G_{ATP}/RT) \qquad \text{(S44)}$$

$$k_{+2} = \frac{k_2 F E_m}{RT} \frac{1}{1-\exp(-FE_m/RT)}$$

$$k_{-2} = \frac{k_2 [Ca^{2+}]_{out} [H^+]_{in} F E_m}{RT} \frac{\exp(-FE_m/RT)}{1-\exp(-FE_m/RT)}$$

where $[Ca^{2+}]_{out}$ and $[Ca^{2+}]_{in}$ are concentrations of calcium ions in apoplast and cytoplasm, respectively; $[H^+]_{out}$ and $[H^+]_{in}$ are concentrations of protons in apoplast and cytoplasm, respectively; k_1 (28.35 s^{-1}) and k_2 (1.62779×10^{-5} s^{-1}) are velocity constants at E_m=0 mV and $[Ca^{2+}]_{out} = [Ca^{2+}]_{in} = [H^+]_{out} = [H^+]_{in}$ = 1 M; ΔG_{ATP}=-50 000 J mol^{-1} is the energy of the ATP hydrolysis; F (96 500 C mol^{-1}), R (8.31 J K^{-1}), and T are standard thermodynamic parameters.

I^T is described by equation (S45):

$$I^T = Q_{10}^{\frac{T_0 - T}{10}} \qquad \text{(S45)}$$

where Q_{10} (3) is the temperature coefficient of the Ca^{2+}-ATP-ase, T_0 (298 K) is initial temperature.

Equation (S41) is used for calculation of i_P^{Ca}:

$$i_P^{Ca} = zFj_P^{Ca} \qquad \text{(S46)}$$

where z (+1) is charge, which is transferred per 1 enzyme turnover.

Equation (S47) is used for calculation of E_P^H:

$$E_P^H = \frac{\Delta G_{ATP}}{F} + \frac{RT}{F} \ln\left(\frac{[Ca^{2+}]_{out}}{[Ca^{2+}]_{in}} \frac{[H^+]_{in}}{[H^+]_{out}} \right) \qquad \text{(S47)}$$

2.9. $2H^+$-Cl^- antiporter

Ion flux from the $2H^+$-Cl^- antiporter in the plasma membrane (j_P^{Cl}) is described by equation (S48):

$$j_P^{Cl} = I^T V^{Cl} \frac{FE_m/RT}{1-\exp(-FE_m/RT)} \left([Cl^-]_{in} [H^+]_{in}^2 - [Cl^-]_{out} [H^+]_{out}^2 \exp(-FE_m/RT) \right) \qquad \text{(S48)}$$

where V^{Cl} is parameter which is proportional to rate of transports of ions through the antiporter (20000 $s^{-1}M^{-2}$); I^T is variable describing regulation of activities of the $2H^+$-Cl^- antiporter by temperature (T); $[Cl^-]_{out}$ and $[Cl^-]_{in}$ are concentrations of chlorine ions in apoplast and cytoplasm, respectively; $[H^+]_{out}$ and $[H^+]_{in}$ are concentrations of protons in apoplast and cytoplasm, respectively; F (96 500 C mol^{-1}), R (8.31 J K^{-1}), and T are standard thermodynamic parameters.

I^T is described by equation (S49):

$$I^T = Q_{10}^{\frac{T_0 - T}{10}} \tag{S49}$$

where Q_{10} (3) is the temperature coefficient of the $2H^+$-Cl^- antiporter, T_0 (298 K) is initial temperature.

Equation (S50) is used for calculation of i_P^{Cl}:

$$i_P^{Cl} = zFj_P^{Cl} \tag{S50}$$

where z (+1) is charge, which is transferred per 1 enzyme turnover.

Equation (S51) is used for calculation of E_P^{Cl}:

$$E_P^{Cl} = \frac{RT}{F}\ln\left(\frac{[Cl^-]_{out}}{[Cl^-]_{in}}\frac{[H^+]_{out}^2}{[H^+]_{in}^2}\right) \tag{S51}$$

2.10. H^+-K^+ symporter

Ion flux from the H^+-K^+ symporter in the plasma membrane (j_p^K) is described by equation (S52):

$$j_P^K = I^T V^K\left([K^+]_{in}[H^+]_{out} - [K^+]_{out}[H^+]_{in}\right) \tag{S52}$$

where V^K is parameter which is proportional to rate of transports of ions through the symporter (0.015 $s^{-1}M^{-1}$); I^T is variable describing regulation of activities of the H^+-K^+ symporter by temperature (T); $[K^+]_{out}$ and $[K^+]_{in}$ are concentrations of potassium ions in apoplast and cytoplasm, respectively; $[H^+]_{out}$ and $[H^+]_{in}$ are concentrations of protons in apoplast and cytoplasm, respectively.

I^T is described by equation (S53):

$$I^T = Q_{10}^{\frac{T_0 - T}{10}} \tag{S53}$$

where Q_{10} (3) is the temperature coefficient of the H^+-K^+ symporter, T_0 (298 K) is initial temperature.

3. Descriptions of changes in concentrations of ions

3.1. Descriptions of ion buffers in cytoplasm and apoplast

Equation (S54) describes dependence of H^+ concentration in cytoplasm ($[H^+]_{in}$) on total concentration of protons in the cytoplasm ($[H]_{in}$):

$$[H^+]_{in} = \frac{[H]_{in} - B_{in}^0 - K_d^{BHin}}{2} + \sqrt{\left(\frac{[H]_{in} - B_{in}^0 - K_d^{BHin}}{2}\right)^2 + K_d^{BHin}[H]_{in}} \tag{S54}$$

where B_{in}^0 (0.2 M) is the total concentration of the proton buffer in cytoplasm; K_d^{BHin} (10^{-6} M) is the constant of dissociation between the proton and molecule of the buffer.

Equation (S55) describes dependence of H^+ concentration in apoplast ($[H^+]_{out}$) on total concentration of protons in the apoplast ($[H]_{out}$) and equation (S56) describes dependence of K^+

concentration in apoplast ($[K^+]_{out}$) on total concentration of potassium ions in the apoplast ($[K]_{out}$).

$$[H^+]_{in} = \frac{[H]_{out} + [K]_{out} - B_{out}{}^0 - K_d{}^{BHout}}{2} \frac{[H]_{out} K_d{}^{BHout}}{[H]_{out} K_d{}^{BHout} + [K]_{out} K_d{}^{BKout}} + \tag{S55}$$

$$+ \sqrt{\left(\frac{[H]_{out} + [K]_{out} - B_{out}{}^0 - K_d{}^{BHout}}{2} \frac{[H]_{out} K_d{}^{BHout}}{[H]_{out} K_d{}^{BHout} + [K]_{out} K_d{}^{BKout}} \right)^2 + \frac{\left([H]_{out} K_d{}^{BHout}\right)^2}{[H]_{out} K_d{}^{BHout} + [K]_{out} K_d{}^{BKout}}}$$

$$[K^+]_{in} = [H^+]_{out} \frac{[K]_{out} K_d{}^{BKout}}{[H]_{out} K_d{}^{BHout}} \tag{S56}$$

where $B_{out}{}^0$ (0.0833 M) is the total concentration of the proton and potassium buffer in the apoplast; $K_d{}^{BHout}$ (10^{-6} M) and $K_d{}^{BKout}$ (10^{-4} M) are the constants of dissociation between the proton and molecule of the buffer and between the potassium and molecule of the buffer, respectively. Equations (S55) and (S56) are correct at assumption that $\frac{\left([K]_{out} - [K^+]_{out}\right) K_d{}^{BKout}}{\left([H]_{out} - [H^+]_{out}\right) K_d{}^{BHout}} \approx \frac{[K]_{out} K_d{}^{BKout}}{[H]_{out} K_d{}^{BHout}}$ (i.e. $[H^+]_{out} << [H]_{out}$ and $[K^+]_{out} << [K]_{out}$).

3.2. Descriptions of changes in concentrations of ions caused by activities of transporters in the plasma membrane

The descriptions of changes in concentrations of ions are based on equations (S57)-(S63).

$$\frac{d[Ca^{2+}]_{in}}{dt} = -\frac{S}{V_{in}} \left(j_C{}^{Ca} + j_P{}^{Ca} \right) \tag{S57}$$

where S/V_{in} (10^5 m^{-1}) is ratio between cell surface (S) and intracellular volume (V_{in}).

$$\frac{d[K^+]_{in}}{dt} = \frac{S}{V_{in}} \left(j_{inC}{}^K + j_{outC}{}^K + j_P{}^K \right) \tag{S58}$$

$$\frac{d[K]_{out}}{dt} = -\frac{S}{V_{in}} \frac{V_{in}}{V_{out}} \left(j_{inC}{}^K + j_{outC}{}^K + j_P{}^K \right) \tag{S59}$$

where V_{in}/V_{out} (0.41667) is ratio between the intracellular (V_{in}) and extracellular (V_{out}) volumes.

$$\frac{d[Cl^-]_{in}}{dt} = \frac{S}{V_{in}} \left(j_C{}^{Cl} + j_P{}^{Cl} \right) \tag{S60}$$

$$\frac{d[Cl^-]_{out}}{dt} = -\frac{S}{V_{in}} \frac{V_{in}}{V_{out}} \left(j_C{}^{Cl} + j_P{}^{Cl} \right) \tag{S61}$$

$$\frac{d[H]_{in}}{dt} = \frac{S}{V_{in}} \left(j_C{}^H + j_P{}^H + 2 j_P{}^{Cl} - j_P{}^{Ca} - j_P{}^K \right) \tag{S62}$$

$$\frac{d[H]_{out}}{dt} = -\frac{S}{V_{in}}\frac{V_{in}}{V_{out}}\left(j_C{}^{H} + j_P{}^{H} + 2j_P{}^{Cl} - j_P{}^{Ca} - j_P{}^{K}\right) \quad \text{(S63)}$$

We assume that Ca^{2+} concentration in apoplast is approximately constant (5×10^{-4} M).

3.3. Descriptions of changes in concentrations of ions caused by their diffusions between apoplast regions

The descriptions of changes in concentrations of ions are based on the Fick's law. Equation (S64) is used:

$$\frac{d[r]_{out}{}^{ij}}{dt} = \frac{D_r}{a^2\left(1 + V_{in}/V_{out}\right)}\left(4[r]_{out}{}^{ij} - [r]_{out}{}^{i-1j} - [r]_{out}{}^{i+1j} - [r]_{out}{}^{ij-1} - [r]_{out}{}^{ij+1}\right) \quad \text{(S64)}$$

where $r \in \left[K^+, Cl^-, H^+\right]$;$[r]_{out}{}^{ij}$ is concentration of ion r in cell *ij*; $[r]_{out}{}^{i-1j}$, $[r]_{out}{}^{i+1j}$, $[r]_{out}{}^{ij-1}$, and $[r]_{out}{}^{ij+1}$ are the ion concentrations in 4 neighboring cells; D_r is coefficient of diffusion for ion r (1.96×10^{-9} m^2s^{-1} for K^+, 2.03×10^{-9} m^2s^{-1} for Cl^-, 7.8×10^{-9} m^2s^{-1} for H^+); a (10^{-4} m) is the linear dimension of the single element of simulated cell array.

4. Initial values of variables in the model

It is assumed that initial $[Ca^{2+}]_{in}$, $[Cl^-]_{in}$, $[Cl^-]_{out}$, $[K^+]_{in}$, $[K]_{out}$, $[H]_{in}$, and $[H]_{out}$ are 10^{-7} M, 2×10^{-2} M, 2.7×10^{-3} M, 0.165 M, 0.082 M (i.e. $[K^+]_{out}=3.5\times10^{-3}$ M), 0.015 M (i.e. $[H^+]_{in}=7\times10^{-8}$ M), and 2.1×10^{-3} M (i.e. $[H^+]_{out}=10^{-6}$ M), respectively. Initial probabilities of open and inactivated state of the all simulated ion channels are calculated as stationary values.

5. Numerical solution of equations of the model

The model equations have been numerically calculated by Euler's method using the computer program (Borland Delphi 7) which was developed for solution of this task.

For acceleration of numerical analysis, the program uses three different time steps (Δt) for three group of processes with different times. Δt = 100 ms is the basic time step, which is used for the processes in the single cell without intercellular interactions, Δt = 25 μs is the time step, which is used for description of the passive membrane potential change propagation, and Δt = 10 ms is the time step, which is used for description of the ion diffusion between apoplast regions of neighboring cells.

The model includes stochastic variable; as a result, the Monte Carlo method is used for simulation (5 or 25 repetitions are used in different variants of analysis). Means, standard errors and significances are calculated for investigated parameters.
